# Coagulopathy in Beta-Thalassemia: Current Understanding and Future Perspectives

**DOI:** 10.4084/MJHID.2009.029

**Published:** 2009-12-29

**Authors:** M. Domenica Cappellini, Khaled M. Musallam, Alessia Marcon, Ali T. Taher

**Affiliations:** 1 Universitá di Milano, Policlinico Foundation IRCCS, Milan, Italy; 2 Department of Internal Medicine, Hematology-Oncology Division, American University of Beirut Medical Centre, Beirut, Lebanon

## Abstract

As the life expectancy of β-thalassemia patients has markedly improved over the last decade, several new complications are being recognized. The presence of a high incidence of thromboembolic events, mainly in thalassemia intermedia patients, has led to the identification of a hypercoagulable state in thalassemia. In this review, the molecular and cellular mechanisms leading to hypercoagulability in thalassemia are highlighted, and the current clinical experience is summarized. Recommendations for thrombosis prophylaxis are also discussed.

## Introduction:

Although once considered a fatal disease, the life expectancy of thalassemia major (TM) patients has markedly improved over the last few years, as a result of regular blood transfusions and compliance with tight iron chelation therapy.^1^ However, TM patients still suffer from many complications of their chronic disease, and a series of serious previously undescribed complications are now being acknowledged, including thrombosis.^2^ Patients with thalassemia intermedia (TI) have, in general, a milder clinical phenotype than those with TM and remain largely transfusion independent^3^. The pathophysiology of TI is characterized by extravascular hemolysis, with the release into the peripheral circulation of damaged red blood cells (RBCs) and erythroid precursors because of a high degree of ineffective erythropoiesis^2^. This has also been recently attributed to severe complications such as pulmonary hypertension (PHT) and thromboembolic phenomena.^2^ This review summarises current knowledge of the clinical and pathophysiological characteristics of hypercoagulability in thalassemia patients and highlights available strategies to prevent the associated thromboemoblic events (TEE).

## Pathogenesis:

**Figure 1.**
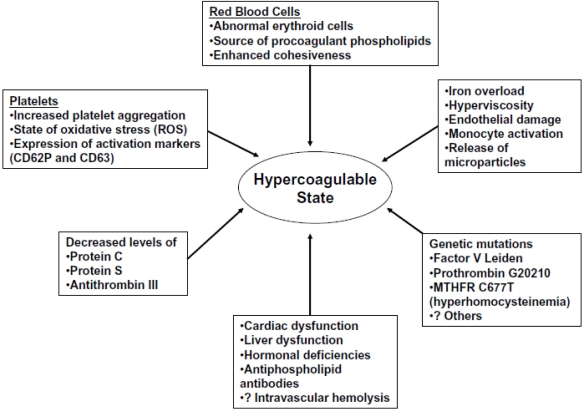
Factors contributing to hypercoagulability in thalassemia (RBCs = red blood cells)^2^.

Guided by clinical observation, diverse factors contributing to the hypercoagulable state in patients with thalassemia have been identified (Figure 1).^2^ In most cases, a combination of these abnormalities leads to clinical thrombosis. Among cellular factors*, platelet activation* contributes to a significant extent. The medical literature is rich in evidence suggesting that patients with thalassemia have activated platelets. Moreover, flow cytometric studies have also confirmed the chronic platelet activation status. In thalassemia, there is evidence of increased platelet aggregation,^4^ an increased proportion of platelets expressing CD62P (P-selectin) and CD63,^5^–^6^ and a shortened platelet survival due to enhanced platelet consumption (especially in splenectomized patients).^7^–^8^

Alteration in *RBCs*, namely the oxidation of globin subunits in thalassemia erythroid cells, leads to the formation of hemichromes. Hemichromes bind to or modify various components of the mature RBC membrane, such as protein band 3, ankyrin, and spectrin. After the precipitation of hemichromes, heme disintegrates, and toxic nontransferrin-bound iron species are released from the heme disintegration. The resulting free iron catalyzes the formation of reactive oxygen species. Iron-dependent oxidation of membrane proteins and formation of red-cell “senescence” antigens such as phosphatidylserine cause thalassemic red cells to be rigid and deformed and to aggregate, resulting in premature cell removal.^9^–^13^ Studies have shown that thalassemic RBCs may be a source of negatively charged phospholipids, which can eventually increase thrombin generation.^14^–^15^ This was verified by experiments that showed that annexin V, a protein with high affinity and specificity for anionic phospholipids, could block the procoagulant effect of isolated thalassemic RBCs.^15^–^16^ Several studies have demonstrated that RBCs from thalassemic patients also show enhanced cohesiveness and aggregability. These abnormalities have been reduced to normal range after the patients have received a blood transfusion.^15^

The finding of elevated levels of *endothelial* adhesion proteins (E-selectin [ELAM-1], intercellular adhesion molecule-1 [ICAM-1] and von Willebrand factor [VWF]) and vascular cell adhesion molecule-1 [VCAM-1] in thalassemic patients suggested that endothelial injury or activation may be a feature of this genetic disease which also plays an important role in the recruitment of white blood cells and RBCs and promote thrombosis at vascular inflammation sites, vessel obstruction, tissue hypoxia and death^17^–^22^. More recently, it was shown that *microparticles* of red blood cell origins were elevated in patients with TI vs. controls; these have a potential to aggravate thrombotic events.^23^

Clinical observations have suggested that splenectomy in TI can contribute to an increased susceptibility to thrombosis.^24^–^26^ The development of these complications has been ascribed to the presence of high platelet counts following splenectomy and/or to increased number of abnormal RBCs.^27^–^29^ In splenectomized TI patients, thrombin generation was significantly higher than in control subjects and patients who had not undergone splenectomy.^26^ From the available data, DNA mutations do not appear to play an important role in the pathogenesis of thrombosis observed in thalassemia. In two studies from the Eastern Mediterranean region the presence of factor V Leiden, prothrombin mutation, and methylene tetrahydrofolate reductase (MTHFR) mutations was not significantly correlated with the thrombotic risk.^30^–^31^ However, many investigators have reported changes in the levels of coagulation factors and inhibitors in thalassemic patients. Prothrombin fragment 1.2 (F1.2), a marker of thrombin generation, is elevated in TI patients. The status of protein C and protein S was investigated in thalassemia in many studies and generally they were found to be decreased; this might be responsible for the occurrence of TEE in thalassemic patients.^26^ The presence of anti-phospholipid antibodies (aPL) has been reported in the serum of thalassemia patients. However, the exact nature of these antibodies and their relation to coexistent hepatitis C virus (HCV) infection is still under investigation.^32^ Other pathogenetic mechanisms have been correlated with hypercoagulability in thalassemia and these include cardiac dysfunction, hormonal deficiencies and liver dysfunction.^2^

The pathophysiological roles of hemolysis and the dysregulation of nitric oxide homeostasis are correlated with pulmonary hypertension in sickle cell disease and in thalassemia. Nitric oxide binds soluble guanylate cyclase, which converts GTP to cGMP, relaxing vascular smooth muscle and causing vasodilatation. When plasma hemoglobin liberated from intravascularly hemolyzed sickle erythrocytes consumes nitric oxide, the balance is shifted toward vasoconstriction. Pulmonary hypertension is aggravated and in sickle cell disease, it is linked to the intensity of hemolysis. Whether the same mechanism contributes to hypercoagulability in thalassemia is not yet known and needs to be investigated.^33^

## Clinical Impact:

There are relatively few epidemiological data on the overall frequency of TEE in patients with thalassemia (Table 1). The largest clinical study to date^25^ analyzed data from 8860 thalassemia patients (6670 TM and 2190 TI). The authors demonstrated that TEE occurred 4.38 times more frequently in TI than TM (p < 0.001), with more venous events occurring in TI and more arterial events occurring in TM. Moreover, patients with TI who developed a TEE were mostly splenectomized, non-transfused, and had a haemoglobin level below 9 g/dl. The study described age beyond 20 years, splenectomy, family history of TEE and previous TEE as the main risk factors for developing thrombosis in the study group.^25^ In another series of TI patients, 24 patients (29%) developed either deep vein thrombosis (DVT), pulmonary embolism, or portal vein thrombosis during a 10-year follow up.^26^ All patients except one had undergone splenectomy. A study on survival and causes of death in TM, carried out in Italy at the end of the 1980s, indicated TEE as the primary cause of death in four of 159 (2.5%) transfusion-dependent thalassemic patients.^34^ In a recent survey involving nine Italian pediatric thalassemia centers, TEE was observed in 4% of 683 patients with TM and in 9.6% of 52 patients with TI.^35^ Even more recently, data from seven Italian centers on 720 patients with TM, 1.1% of the patients had thrombosis.^1^

Logothetis et al. described a “stroke syndrome” and neurological deficits compatible with transient ischemic attacks (TIAs) in about 20% of 138 cases of TM in Greece.^36^ Similarly, Borgna Pignatti et al. described TIAs accompanied by a clinical picture of headache, seizures, and hemiparesis in 2.2% of TM patients in Italy.^35^ Although the incidence of overt stroke in TM was usually described as higher than TI,^25^ a study done to assess the rate of silent brain damage in patients with benign hemoglobinopathies reported that 37.5% of patients with TI showed asymptomatic brain damage on brain magnetic resonance imaging (MRI)^37^. More recently, a brain MRI study on adult, splenectomized TI patients showed a rate of silent white matter lesions as high as 60%.^24^ Older age and transfusion naivety were associated with a higher incidence and multiplicity of lesions.^24^

Autopsy series in patients with TM and TI describe the presence of DVT, pulmonary embolism and recurrent arterial occlusion, with thrombi in small and large pulmonary vessels.^34^,^38^–^39^ Autopsies of a large series of patients with Beta-thalassemia/hemoglobin E disease revealed thrombotic lesions in the pulmonary arteries.^40^ These pulmonary arterial thromboembolism may have been due to circulating platelet aggregates. Similar findings of multiple microthrombi, which were composed mainly of platelets, were seen in the pulmonary arterioles and microcirculation in autopsies of two splenectomized patients with thalassemia.^41^ The aforementioned collective evidence allowed the identification of TEE as an established complication of thalassemia, which is now referred to as a ‘hypercoagulable state’.^42^

## Recommendations for Management:

The higher rate of thrombosis in transfusion-independent TI compared to polytransused TM patients suggests a potential role for transfusions in decreasing the rate of TEE.^24^–^26^,^35^ The reduction of TEE in adequately transfused patients may be the result of decreased numbers of pathological RBCs exhibiting indices of membrane damage.^16^ It should be noted that the benefit of regular blood transfusions is appreciated in the more frequent thromboembolic manifestations in less developed countries with inadequate transfusion resources. Moreover, the higher rate of TEE in splenectomized patients may alter the risk-benefit assessment of splenectomy as a procedure of choice. The available data on the use of anticoagulants, antiplatelet, or other agents in thalassemia are either lacking or involve small, poorly controlled and/or relatively low-quality studies.^2^ However, TI patients who experienced a TEE and received aspirin afterwards had a lower recurrence of TEE compared with those who were not taking aspirin, although these differences were not statistically significant.^25^

Treatment with the fetal hemoglobin-inducing agents, hydroxycarbmide and decitabine, results decreased plasma markers of thrombin generation. Hydroxycarbamide, specifically approved for the treatment of sickle cell disease, may decrease coagulation activation by reducing phospholipid expression on the surface of both RBCs and platelets and decreasing RBC adhesion to thrombospondin. In addition to being a nitric oxide donor, hydroxycarbamide may also decrease hemostatic activation by its effect in decreasing the white blood cell count and particularly monocytes that express transcription factor^33^. Hydroxycarbamide is only rarely used in thalassemia^43^, these patients may experience the benefits because of similar mechanisms described in sickle cell disease. Another approach would be to correct the reactive oxygen species-induced RBC membrane damage using antioxidants, although this approach has not yet been verified in clinical trials.

It may also be possible to design a thalassemia-tailored thrombosis risk-assessment model (RAM) to estimate thrombotic risk as a function of intrinsic (e.g. thalassemia type and number of circulating RBC) and extrinsic (e.g. infection, surgery, and splenectomy) factors. Moreover, tests for predisposing factors could also be performed, particularly in high-risk patients. If clinically verified, this type of model could serve as a guideline for possible preventative treatment to decrease the incidence of TEE, which can cause significant morbidity and mortality.^2^ In fact, attempts to identify diagnostic tests that will help identify patients at risk are emerging^44^, with results that are promising towards establishing an evidence based preventive approach.

## Conclusion:

In conclusion, there are diverse factors contributing to the hypercoagulable state observed in patients with thalassemia. In most cases, a combination of these abnormalities leads to clinical thrombosis. The higher incidence of thrombotic events in TI compared to TM patients is mainly attributed to transfusion naivety and splenectomy, both of which promote an underlying procoagulant activity. Although no clear guidelines exist to establish a prophylactic strategy, an individualized approach that takes into consideration all associated risk factors is advisable.

## Figures and Tables

**Table 1.** Prevalence of thromboembolic events in patients with thalassemia major and intermedia.

**Reference**	**TI n (%)**	**TM n (%)**	**Type of thrombosis**			
			**VT**	**PE**	**AT**	**Stroke**
Michaeli et al, 1992^38^	-	4/100 (4)	*	*	*	*
Aessopos J et al, 1997^45^	3/5 (60)	3/5 (60)				*
Moratelli et al, 1998^46^	12/74 (16.2)	14/421 (3.3)		N/A		
Borgna Pignatti et al, 1998^35^	5/52 (9.6)	27/683 (4.0)	*	*	*	*
Cappellini et al, 2000^26^	24/83 (29)	-	*	*	*	
Taher et al, 2006^25^	85/2190 (3.9)	61/6670 (0.9)	*	*	*	*

TI = thalassemia intermedia, TM = thalassemia major, VT = venous thrombosis, PE = pulmonary embolism, AT = arterial thrombosis, N/A = not available.

## References

[1] Borgna-Pignatti C Rugolotto S De Stefano P Zhao H Cappellini MD Del Vecchio GC Romeo MA Forni GL Gamberini MR Ghilardi R Piga A Cnaan A Survival and complications in patients with thalassemia major treated with transfusion and deferoxamine Haematologica 2004 89 1187 1193 15477202

[2] Taher AT Otrock ZK Uthman I Cappellini MD Thalassemia and hypercoagulability Blood Rev 2008 22 283 292 18511163 10.1016/j.blre.2008.04.001

[3] Taher A Isma’eel H Cappellini MD Thalassemia intermedia: revisited Blood Cells Mol Dis 2006 37 12 20 16737833 10.1016/j.bcmd.2006.04.005

[4] Winichagoon P Fucharoen S Wasi P Increased circulating platelet aggregates in thalassaemia Southeast Asian J Trop Med Public Health 1981 12 556 560 7344105

[5] Del Principe D Menichelli A Di Giulio S De Matteis W Cianciulli P Papa G PADGEM/GMP-140 expression on platelet membranes from homozygous beta thalassaemic patients Br J Haematol 1993 84 111 117 7687856 10.1111/j.1365-2141.1993.tb03033.x

[6] Ruf A Pick M Deutsch V Patscheke H Goldfarb A Rachmilewitz EA Guillin MC Eldor A In-vivo platelet activation correlates with red cell anionic phospholipid exposure in patients with beta-thalassaemia major Br J Haematol 1997 98 51 56 9233563 10.1046/j.1365-2141.1997.1502965.x

[7] Eldor A Krausz Y Atlan H Snyder D Goldfarb A Hy-Am E Rachmilewitz EA Kotze HF Heyns AD Platelet survival in patients with beta-thalassemia Am J Hematol 1989 32 94 99 2757016 10.1002/ajh.2830320204

[8] Eldor A Lellouche F Goldfarb A Rachmilewitz EA Maclouf J In vivo platelet activation in beta-thalassemia major reflected by increased platelet-thromboxane urinary metabolites Blood 1991 77 1749 1753 2015401

[9] Rund D Rachmilewitz E Beta-thalassemia N Engl J Med 2005 353 1135 1146 16162884 10.1056/NEJMra050436

[10] Shinar E Rachmilewitz EA Lux SE Differing erythrocyte membrane skeletal protein defects in alpha and beta thalassemia J Clin Invest 1989 83 404 410 2521488 10.1172/JCI113898 PMC303695

[11] Hershko C Graham G Bates GW Rachmilewitz EA Non-specific serum iron in thalassaemia: an abnormal serum iron fraction of potential toxicity Br J Haematol 1978 40 255 263 708645 10.1111/j.1365-2141.1978.tb03662.x

[12] Kuypers FA de Jong K The role of phosphatidylserine in recognition and removal of erythrocytes Cell Mol Biol (Noisy-le-grand) 2004 50 147 158 15095785

[13] Tavazzi D Duca L Graziadei G Comino A Fiorelli G Cappellini MD Membrane-bound iron contributes to oxidative damage of beta-thalassaemia intermedia erythrocytes Br J Haematol 2001 112 48 50 11167782 10.1046/j.1365-2141.2001.02482.x

[14] Borenstain-Ben Yashar V Barenholz Y Hy-Am E Rachmilewitz EA Eldor A Phosphatidylserine in the outer leaflet of red blood cells from beta-thalassemia patients may explain the chronic hypercoagulable state and thrombotic episodes Am J Hematol 1993 44 63 65 8342566 10.1002/ajh.2830440114

[15] Helley D Eldor A Girot R Ducrocq R Guillin MC Bezeaud A Increased procoagulant activity of red blood cells from patients with homozygous sickle cell disease and beta-thalassemia Thromb Haemost 1996 76 322 327 8883264

[16] Chen S Eldor A Barshtein G Zhang S Goldfarb A Rachmilewitz E Yedgar S Enhanced aggregability of red blood cells of beta-thalassemia major patients Am J Physiol 1996 270 H1951 1956 8764243 10.1152/ajpheart.1996.270.6.H1951

[17] Butthep P Bunyaratvej A Funahara Y Kitaguchi H Fucharoen S Sato S Bhamarapravati N Alterations in vascular endothelial cell-related plasma proteins in thalassaemic patients and their correlation with clinical symptoms Thromb Haemost 1995 74 1045 1049 8560411

[18] Butthep P Bunyaratvej A Funahara Y Kitaguchi H Fucharoen S Sato S Bhamarapravati N Possible evidence of endothelial cell activation and disturbance in thalassemia: an in vitro study Southeast Asian J Trop Med Public Health 1997 28 Suppl 3 141 148A 9640617

[19] Hovav T Goldfarb A Artmann G Yedgar S Barshtein G Enhanced adherence of beta-thalassaemic erythrocytes to endothelial cells Br J Haematol 1999 106 178 181 10444184 10.1046/j.1365-2141.1999.01489.x

[20] Butthep P Rummavas S Wisedpanichkij R Jindadamrongwech S Fucharoen S Bunyaratvej A Increased circulating activated endothelial cells, vascular endothelial growth factor, and tumor necrosis factor in thalassemia Am J Hematol 2002 70 100 106 12111782 10.1002/ajh.10101

[21] Carlos TM Harlan JM Leukocyte-endothelial adhesion molecules Blood 1994 84 2068 2101 7522621

[22] Mann KG van’t Veer C Cawthern K Butenas S The role of the tissue factor pathway in initiation of coagulation Blood Coagul Fibrinolysis 1998 9 Suppl 1 S3 7 9819022

[23] Habib A Kunzelmann C Shamseddeen W Zobairi F Freyssinet JM Taher A Elevated levels of circulating procoagulant microparticles in patients with beta-thalassemia intermedia Haematologica 2008 93 941 942 18460647 10.3324/haematol.12460

[24] Taher AT Musallam KM Nasreddine W Hourani R Inati A Beydoun A Asymptomatic brain magnetic resonance imaging abnormalities in splenectomized adults with thalassemia intermedia J Thromb Haemost 2009 10.1111/j.1538-7836.2009.03651.x 19817994

[25] Taher A Isma’eel H Mehio G Bignamini D Kattamis A Rachmilewitz EA Cappellini MD Prevalence of thromboembolic events among 8,860 patients with thalassaemia major and intermedia in the Mediterranean area and Iran Thromb Haemost 2006 96 488 491 17003927

[26] Cappellini MD Robbiolo L Bottasso BM Coppola R Fiorelli G Mannucci AP Venous thromboembolism and hypercoagulability in splenectomized patients with thalassaemia intermedia Br J Haematol 2000 111 467 473 11122086 10.1046/j.1365-2141.2000.02376.x

[27] Atichartakarn V Angchaisuksiri P Aryurachai K Onpun S Chuncharunee S Thakkinstian A Atamasirikul K Relationship between hypercoagulable state and erythrocyte phosphatidylserine exposure in splenectomized haemoglobin E/beta-thalassaemic patients Br J Haematol 2002 118 893 898 12181063 10.1046/j.1365-2141.2002.03711.x

[28] Cappellini MD Grespi E Cassinerio E Bignamini D Fiorelli G Coagulation and splenectomy: an overview Ann N Y Acad Sci 2005 1054 317 324 16339680 10.1196/annals.1345.039

[29] Atichartakarn V Angchaisuksiri P Aryurachai K Chuncharunee S Thakkinstian A In vivo platelet activation and hyperaggregation in hemoglobin E/beta-thalassemia: a consequence of splenectomy Int J Hematol 2003 77 299 303 12731676 10.1007/BF02983790

[30] Iolascon A Giordano P Storelli S Li HH Coppola B Piga A Fantola E Forni G Cianciulli P Perrotta S Magnano C Maggio A Mangiagli A Devoto M Thrombophilia in thalassemia major patients: analysis of genetic predisposing factors Haematologica 2001 86 1112 1113 11602424

[31] Zalloua PA Shbaklo H Mourad YA Koussa S Taher A Incidence of thromboembolic events in Lebanese thalassemia intermedia patients Thromb Haemost 2003 89 767 768 12669135

[32] Giordano P Galli M Del Vecchio GC Altomare M Norbis F Ruggeri L Petronelli M de Mattia D Lupus anticoagulant, anticardiolipin antibodies and hepatitis C virus infection in thalassaemia Br J Haematol 1998 102 903 906 9734637 10.1046/j.1365-2141.1998.00853.x

[33] Ataga KI Cappellini MD Rachmilewitz EA Beta-thalassaemia and sickle cell anaemia as paradigms of hypercoagulability Br J Haematol 2007 139 3 13 17854302 10.1111/j.1365-2141.2007.06740.x

[34] Zurlo MG De Stefano P Borgna-Pignatti C Di Palma A Piga A Melevendi C Di Gregorio F Burattini MG Terzoli S Survival and causes of death in thalassaemia major Lancet 1989 2 27 30 2567801 10.1016/s0140-6736(89)90264-x

[35] Borgna Pignatti C Carnelli V Caruso V Dore F De Mattia D Di Palma A Di Gregorio F Romeo MA Longhi R Mangiagli A Melevendi C Pizzarelli G Musumeci S Thromboembolic events in beta thalassemia major: an Italian multicenter study Acta Haematol 1998 99 76 79 9554453 10.1159/000040814

[36] Logothetis J Constantoulakis M Economidou J Stefanis C Hakas P Augoustaki O Sofroniadou K Loewenson R Bilek M Thalassemia major (homozygous beta-thalassemia). A survey of 138 cases with emphasis on neurologic and muscular aspects Neurology 1972 22 294 304 5062264 10.1212/wnl.22.3.294

[37] Manfre L Giarratano E Maggio A Banco A Vaccaro G Lagalla R MR imaging of the brain: findings in asymptomatic patients with thalassemia intermedia and sickle cell-thalassemia disease AJR Am J Roentgenol 1999 173 1477 1480 10584785 10.2214/ajr.173.6.10584785

[38] Michaeli J Mittelman M Grisaru D Rachmilewitz EA Thromboembolic complications in beta thalassemia major Acta Haematol 1992 87 71 74 1585774 10.1159/000204720

[39] Gillis S Cappellini MD Goldfarb A Ciceri L Fiorelli G Rachmilewitz EA Pulmonary thromboembolism in thalassemia intermedia patients Haematologica 1999 84 959 960 10509051

[40] Sonakul D Pacharee P Laohapand T Fucharoen S Wasi P Pulmonary artery obstruction in thalassaemia Southeast Asian J Trop Med Public Health 1980 11 516 523 7221695

[41] Sumiyoshi A Thakerngpol K Sonakul D Pulmonary microthromboemboli in thalassemic cases Southeast Asian J Trop Med Public Health 1992 23 Suppl 2 29 31 1298989

[42] Eldor A Rachmilewitz EA The hypercoagulable state in thalassemia Blood 2002 99 36 43 11756150 10.1182/blood.v99.1.36

[43] Karimi M Darzi H Yavarian M Hematologic and clinical responses of thalassemia intermedia patients to hydroxyurea during 6 years of therapy in Iran J Pediatr Hematol Oncol 2005 27 380 385 16012328 10.1097/01.mph.0000174386.13109.28

[44] Tripodi A Cappellini MD Chantarangkul V Padovan L Fasulo MR Marcon A Mannucci PM Hypercoagulability in splenectomized thalassemic patients detected by whole-blood thromboelastometry, but not by thrombin generation in platelet-poor plasma Haematologica 2009 94 1520 1527 19648162 10.3324/haematol.2009.010546 PMC2770962

[45] Aessopos A Farmakis D Karagiorga M Rombos I Loucopoulos D Pseudoxanthoma elasticum lesions and cardiac complications as contributing factors for strokes in beta-thalassemia patients Stroke 1997 28 2421 2424 9412625 10.1161/01.str.28.12.2421

[46] Moratelli S De Sanctis V Gemmati D Serino ML Mari R Gamberini MR Scapoli GL Thrombotic risk in thalassemic patients J Pediatr Endocrinol Metab 1998 11 Suppl 3 915 921 10091165

